# Supplementary material for: Obesity and Associated Factors in Brazilian Adults: Systematic Review and Meta-Analysis of Representative Studies
Source: Int J Environ Res Public Health. 2024 Aug 2;21(8):1022. doi: 10.3390/ijerph21081022 (PMC11354148; doi:10.3390/ijerph21081022)
Supplement: Supplementary file 1 [file ijerph-21-01022-s001.zip › Supplementary Material-S1.pdf]

S1. Search Strategy - Table, describing the methodology of primary population-based studies

PubMed

("obesity"[MeSH Terms] AND ("prevalence"[MeSH Terms] OR "Prevalences"[All Fields] OR "Period Prevalence"[All Fields] OR "Period Prevalences"[All Fields] OR "prevalence period"[All Fields] OR "Point Prevalence"[All Fields] OR "Point Prevalences"[All Fields] OR "prevalence point"[All Fields]) AND "brazil"[MeSH Terms]) AND (2000:2023[pdat])

Embase

		Results
#6	#1 AND #2 AND #3 AND #4 AND (2000:py OR 2001:py OR 2002:py OR 2003:py OR 2004:py OR 2005:py OR 2006:py OR 2007:py OR 2008:py OR 2009:py OR 2010:py OR 2011:py OR 2012:py OR 2013:py OR 2014:py OR 2015:py OR 2016:py OR 2017:py OR 2018:py OR 2019:py OR 2020:py OR 2021:py OR 2022:py)	1,400
#5	#1 AND #2 AND #3 AND #4	1,404
#4	embase NOT 'medline'	9,042,459
#3	'brazil'/exp OR 'brazil'/syn	771,410
#2	'prevalence'/exp OR 'prevalence'/syn	1,326,814
#1	'obesity'/exp OR 'obesity'/syn	769,527

LILACS

tw:((tw:("obesity" OR "obesity" OR "obesidad")) AND (tw:("prevalência" OR "prevalence" OR "prevalencia")) AND (tw:("brasil" OR "brazil"))) AND (collection:("06-national/BR" OR "05-specialized") OR db:("LILACS" OR "MEDLINE")) AND (db:("LILACS"))

Scopus

(TITLE-ABS-KEY (obesity) AND TITLE-ABS-KEY (prevalence OR prevalences) AND TITLE-ABS-KEY (brazil)) AND NOT (pubmed) AND NOT (embase) AND (LIMIT-TO (AFFILCOUNTRY , "Brazil")) AND (LIMIT-TO (DOCTYPE , "ar")) AND (LIMIT-TO (PUBYEAR , 2023) OR LIMIT-TO (PUBYEAR , 2022) OR LIMIT-TO (PUBYEAR , 2021) OR LIMIT-TO (PUBYEAR , 2020) OR LIMIT-TO (PUBYEAR , 2019) OR LIMIT-TO (PUBYEAR , 2018) OR LIMIT-TO (PUBYEAR , 2017) OR LIMIT-TO (PUBYEAR , 2016) OR LIMIT-TO (PUBYEAR , 2015) OR LIMIT-TO (PUBYEAR , 2014) OR LIMIT-TO (PUBYEAR , 2013) OR LIMIT-TO (PUBYEAR , 2012) OR LIMIT-TO (PUBYEAR , 2011) OR LIMIT-TO (PUBYEAR , 2010) OR LIMIT-TO (PUBYEAR , 2009) OR LIMIT-TO (PUBYEAR , 2008) OR LIMIT-TO (PUBYEAR , 2007) OR LIMIT-TO (PUBYEAR , 2006) OR LIMIT-TO (PUBYEAR , 2005) OR LIMIT-TO (PUBYEAR , 2004) OR LIMIT-TO (PUBYEAR , 2003) OR LIMIT-TO (PUBYEAR , 2002) OR LIMIT-TO (PUBYEAR , 2001) OR LIMIT-TO (PUBYEAR , 2000)) AND (LIMIT-TO (SUBJAREA , "MEDI") OR LIMIT-TO (SUBJAREA , "HEAL"))
