# Supplementary material for: Obesity and Associated Factors in Brazilian Adults: Systematic Review and Meta-Analysis of Representative Studies
Source: Int J Environ Res Public Health. 2024 Aug 2;21(8):1022. doi: 10.3390/ijerph21081022 (PMC11354148; doi:10.3390/ijerph21081022)
Supplement: Supplementary file 1 [file ijerph-21-01022-s001.zip › Supplementary Material-S2.pdf]

S2 Table describing the methodology of primary population-based studies

Study	Sample design	Coverage
ENDEF, 1974 [40]	Cluster sampling in two stages (census tract and household)	Brazil, with the exception of the interior of the North region
PNSN, 1989 [41]	Cluster sampling in two stages (census tract and household)	Brazil as a whole
POF, 2008 [42]	Cluster sampling in two stages (census tract and household)	Brazil as a whole
PNS, 2013 [43]	Cluster sampling in three stages (census tract, household, individual ≥18 years old)	Brazil as a whole
PNS, 2019 [44]	Cluster sampling in three stages (census tract, household, individual ≥15 years old)	Brazil as a whole
Vigitel, 2006 [45]	Two-stage cluster sampling (home telephone lines, individual ≥18 years old)[a]	Brazilian capitals and DF
Vigitel, 2007 [46]	Two-stage cluster sampling (home telephone lines, individual ≥18 years old)[a]	Brazilian capitals and DF
Vigitel, 2008 [47]	Two-stage cluster sampling (home telephone lines, individual ≥18 years old)[a]	Brazilian capitals and DF
Vigitel, 2009 [48]	Two-stage cluster sampling (home telephone lines, individual ≥18 years old)[a]	Brazilian capitals and DF
Vigitel, 2010 [49]	Two-stage cluster sampling (home telephone lines, individual ≥18 years old)[a]	Brazilian capitals and DF
Vigitel, 2011 [50]	Two-stage cluster sampling (home telephone lines, individual ≥18 years old)[a]	Brazilian capitals and DF
Vigitel, 2012 [51]	Two-stage cluster sampling (home telephone lines, individual ≥18 years old)[a]	Brazilian capitals and DF
Vigitel, 2013 [52]	Two-stage cluster sampling (home telephone lines, individual ≥18 years old)[a]	Brazilian capitals and DF
Vigitel, 2014 [53]	Two-stage cluster sampling (home telephone lines, individual ≥18 years old)[a]	Brazilian capitals and DF
Vigitel, 2015 [54]	Two-stage cluster sampling (home telephone lines, individual ≥18 years old)[a]	Brazilian capitals and DF
Vigitel, 2016 [55]	Two-stage cluster sampling (home telephone lines, individual ≥18 years old)[a]	Brazilian capitals and DF
Vigitel, 2017 [56]	Two-stage cluster sampling (home telephone lines, individual ≥18 years old)[a]	Brazilian capitals and DF
Vigitel, 2018 [57]	Two-stage cluster sampling (home telephone lines, individual ≥18 years old)[a]	Brazilian capitals and DF
Vigitel, 2019 [58]	Two-stage cluster sampling (home telephone lines, individual ≥18 years old)[a]	Brazilian capitals and DF
Vigitel, 2020 [59]	Two-stage cluster sampling (home telephone lines, individual ≥18 years old)[a]	Brazilian capitals and DF
Vigitel, 2021 [60]	Two-stage cluster sampling (home telephone lines, individual ≥18 years old)[a]	Brazilian capitals and DF

ENDEF, Estudo Nacional de Despesas familiares (National Study of Family Expenses). PNSN, Pesquisa Nacional de Saúde e Nutrição (National Survey on Health and Nutrition). POF, Pesquisa de Orçamentos Familiares (Household Budget Survey). PNS, Pesquisa Nacional de Saúde (National Health Survey). Vigitel, Sistema de Vigilância de Fatores de Risco e Proteção para Doenças Crônicas por Inquérito Telefônico (Surveillance System for Risk and Protection Factors for Chronic Diseases by Telephone Survey). IBGE, Instituto Brasileiro de Geografia e Estatística (Brazilian Statistics Institute);[a] peso pós-

estratificaçãométodo Rake (Graham, 1983) aplicados à amostra Vigitel, igualam sua distribuição sociodemográfica à distribuição estimada para a população total da cidade.
