# Supplementary material for: Obesity and Associated Factors in Brazilian Adults: Systematic Review and Meta-Analysis of Representative Studies
Source: Int J Environ Res Public Health. 2024 Aug 2;21(8):1022. doi: 10.3390/ijerph21081022 (PMC11354148; doi:10.3390/ijerph21081022)
Supplement: Supplementary file 1 [file ijerph-21-01022-s001.zip › Supplementary Material-S3.pdf]

S3. Table Excluded studies from meta-analysis

Reason	First author,year
WRONG OUTCOMES	Sichieri et al.; 2010 [80]
	Conde et al.; 2011 [81]
	Malta et al.; 2014 [82]
	Bernal et al.; 2016 [83]
	Ferreira et al.; 2019 [84]
	Flores-Ortiz et al.; 2019 [85]
	Gomes et al.; 2019 [86]
	Malta et al.; 2019 [87]
	Vale et al.; 2019 [88]
	Wagner et al.; 2019 [89]
	Brebal et al.; 2020 [90]
	Abbade, E.B.; 2021 [91]
	Carvalho et al.; 2022 [92]
WRONG POPULATION	Silva et al.; 2002 [93]
	Batista Filho et al.; 2003 [94]
	Monteiro et al.; 2003 [95]
	Lobato et al.; 2009 [96]
	Costa, L.C.; 2010 [97]
	Lobato et al.; 2015 [98]
	Amann et al.; 2019 [99]
WRONG STUDY DESIGN	Batista Filho et al.; 2007 [100]
	Oliveira et al.; 2013 [101]
	Pavão et al.; 2013 [102]
	Kovalskys et al.; 2016 [103]
	Kudel et al.; 2018 [104]
	Brasil. Ministério da Saúde; 2020 [105]
INCOMPLETE STUDY	Teo et al.; 2010 [106]
